# Psychological Distress in Women with Chronic Bronchitis in a Fishing Community in the Niger Delta Region of Nigeria

**DOI:** 10.1155/2013/526463

**Published:** 2013-12-05

**Authors:** Victor Aniedi Umoh, Andrew Ibok, Bassey Edet, Ekpe Essien, Festus Abasiubong

**Affiliations:** ^1^Department of Internal Medicine, University of Uyo, Uyo, Akwa Ibom State, Nigeria; ^2^Department of Internal Medicine, University of Calabar Teaching Hospital, Nigeria; ^3^Federal Neuropsychiatric Hospital Calabar, Nigeria; ^4^Department of Mental Health, University of Uyo, Nigeria

## Abstract

*Background*. Biomass smoke exposure is a known risk factor for chronic bronchitis. Psychiatric comorbidities may have significant impact on the quality of life of patients with chronic bronchitis. *Methods*. Women who engage in fish preservation by drying over burning firewood in a fishing community were recruited for this survey. The British medical research questionnaire was used to determine chronic bronchitis, and psychological distress was determined using the hospital anxiety and depression scale. *Results*. A total of 342 women were recruited for this study and 63 of them had chronic bronchitis. 96 women had features suggestive of psychological distress: 57 (16.6%) women with anxiety, 51 (14.9%) women with depression and 12 women (3.5%) had combined features. Psychological distress was more common among women with chronic bronchitis. Anxiety was significantly associated with chronic bronchitis and the level of biomass exposure while depression was significantly associated with chronic bronchitis, level of exposure, and a history of sleeping in the fish smoking room. *Conclusion*. Anxiety and depression show significant association with chronic bronchitis among women with biomass smoke exposure with the level of exposure having an aggravating effect on the relationship.

## 1. Introduction 

Chronic obstructive pulmonary disease (COPD) is essentially an irreversible and progressive disease of airflow limitation in the lung caused by small airway disease and parenchyma destruction. It is largely preventable and a major public health concern worldwide [1]. The chronic airflow limitation which is the hallmark of COPD is caused by small airway disease, that is, chronic bronchitis, and the destruction of lung parenchyma, that is, emphysema. Chronic bronchitis is defined clinically as chronic productive cough on most days for three months in each of two successive years in a patient in whom other causes of productive chronic cough have been excluded [2]. Emphysema is defined pathologically as the presence of permanent enlargement of the airspaces distal to the terminal bronchioles, accompanied by the destruction of their walls and without obvious fibrosis [3]. The relative contribution of each of these conditions to an individual patient varies and either of these conditions may or may not occur in COPD.

COPD is the fourth leading cause of death in the United States and is expected to surpass stroke within a decade to be the third leading cause of death [4]. However, in the developed countries, the prevalence of COPD/chronic bronchitis ranges between 3% and 17%, whilst in the developing countries, it ranges between 13% and 27% [5].

In the developed countries, it has been shown that cigarette smoking is the most important risk factor for the development of chronic bronchitis [6]; this explains the much higher prevalence of the illness among males than females as males are more likely to smoke than females. However, in the developing countries, especially in rural populations, where the women are less likely to smoke, studies have shown similar rates of prevalence between the males and females [7]. This similarity in rates may be explained by another contributory risk factor: smoke from domestic biomass fuel consumption. In its 1993 annual report, the World Bank estimated that indoor air pollution is responsible for almost 50% of the total burden of disease resulting from poor household environments in developing countries [8].

Psychiatric comorbidities, including depression, anxiety, and psychosis, have been well documented as significant factors in the morbidity and mortality of COPD patients [9, 10]. COPD has also been shown to be also a risk factor for the development of psychiatric illness [11]. The symptoms of COPD such as dyspnea, exercise intolerance, and inability to fulfill expected social roles may lead to anxiety [12]. Some depressive somatic symptoms like loss of appetite, poor sleep and loss of interest in pleasure activities may mimic symptoms of COPD and can sometimes occur due to an emotional reaction to the distress of coping with COPD [13].

The detection of psychological illness in COPD may be challenging because many physicians are not confident enough to do a thorough psychiatric assessment and may even hesitate to make a clear diagnosis due to the stigma attached to such a diagnosis. Undiagnosed and untreated, anxiety and depressive symptoms may relate to physical disability, impaired quality of life, and increased health care utilization [12].

Few studies in Nigeria have looked at the association between long-term exposure to indoor air pollution and respiratory functions [14, 15]. Fewer still have evaluated this association among rural people exposed to indoor air pollution from fish drying [16, 17] but none of them have examined the psychological distress that may be associated with long-term exposure to indoor air pollution.

This study sought to look at the relationship between psychological distress and chronic bronchitis among women exposed to indoor air pollution from biomass fuel combustion while drying fish.

## 2. Materials and Methods

### 2.1. Study Area

Ibaka is a coastal fishing settlement in Mbo local government area in Akwa Ibom state, in the Niger Delta region of Nigeria. It has an estimated population of 176,680 people by the national population census [18]. It is located south-west of Calabar on the coastal plain and it is assessable by sea and land. The major industry here is fishing and as such most of the inhabitants are engaged in fishing and the processing of the marine products.

### 2.2. Subjects

Three hundred and forty-two women who are engaged in fish processing were recruited for this survey. The fish drying process is usually carried out indoors in drying huts. These huts are constructed with dried mud bricks with a thatch roof. An average drying hut will measure seven by four meters with a door at each end of the room but no windows. The drying area is constructed by pacing a wire mesh or sticks on four wooden supports at a height of about 1.3 meter above the ground. Firewood is burnt beneath the net to produce heat and smoke while the fish and other marine products are placed on the net over the fire. The drying process usually takes a week to complete.

There were over two thousand five hundred houses spread out in eight clusters. Each cluster contained about three hundred houses. The minimum number of subjects required for this survey was determined to be 334 women using the formula *Nf* = *n*/1 + *n*/*N*, where *Nf* is the minimum sample size for a population <10,000. *n* is the minimum sample size where the population is > 10,000 and *N* is the estimated population size of 2,500 (assuming one woman in every household). *n* was found to be 384 (using the following formula *Z* ^2^ *pq*/*d* ^2^). Sixty women were selected from each cluster of houses. A subject was selected from every fourth house by simple ballot and the first house was selected by the same process. Three hundred and fifty-one women agreed to participate in the survey (73% response). Of this number 9 subjects were excluded from the final analysis due to incomplete data.

### 2.3. Ethical Considerations

A signed informed consent was obtained from all participants after careful explanation in the subjects best understood language.

### 2.4. Interview

A questionnaire was designed to collect data from the subjects. It was made up of three parts: the first part consisted of questions related to demography, the second part consisted of the British Medical Council respiratory disease questionnaire [19], and the third part consisted of the hospital anxiety and depression scale (HADS) [20]. The questionnaire was translated into the local language and back translated into English to ensure accuracy. The questionnaire was administered on the subjects by a trained interviewer. It was used to document demographic data and to obtain relevant clinical information. Chronic bronchitis was defined as productive cough on most days for three months in two consecutive years. HADS is specifically designed to identify possible anxiety and depressive symptoms in people. It has been used extensively as both a clinical and research tool. It has been demonstrated to be a reliable and valid instrument for assessing anxiety and depression in patients and has been translated into many languages [21]. HADS has also been validated for use in rural community screening in Nigeria [22]. The HADS consists of 14 items to assess anxiety (seven items) and depression (seven items). Each item is rated from zero to three, with higher scores indicating greater risk for anxiety or depression. The maximum score on either subscale is 21. A score between eight and ten suggests possible or borderline psychological distress while a score above ten suggests probable or definite psychological distress in either domain [20].

### 2.5. Statistical Analysis

Data obtained from the survey was analysed using the Statistical Package for Social Sciences (SPSS) 16.0 computer software. Two groups of women were determined: those with chronic bronchitis and those without. Qualitative data is presented as frequency distributions and cross-tabulations while quantitative data is presented as means and standard deviations. Independent *t*-test was used to compare means between unpaired samples while chi square test was used to test for strength of association between categorical variables. Unadjusted odds ratios (OR) were calculated for the risk of anxiety and depression between the women with and without chronic bronchitis. Direct logistic regression was performed to determine the adjusted OR for the association between chronic bronchitis and demographic variables and psychological distress. A subject with a score of > ten on either anxiety or depression subscale was classified as having either anxiety or depression. Cumulative exposure to biomass smoke among the subjects was given as the product of the average hours per day spent close to the fire and the years spent drying fish (hour-years) as used in previous studies [23]. A *P* value of less than 0.05 was considered to be statistically significant.

## 3. Results

### 3.1. General Characteristics of Subjects

Table 1 shows the characteristics of the study population. Three hundred and forty two women were recruited for this survey, made up of 63 women with chronic bronchitis and 279 without chronic bronchitis. The women with chronic bronchitis were older and had more firewood smoke exposure than those without chronic bronchitis: 42.9 ± 8.8 years versus 39.6 ± 8.9 years and 210.2 ± 14.4 hour-years versus 155.4 ± 6.3 hour-years, respectively (*P* < 0.05). Chronic bronchitis was more prevalent among women who slept in the same room where the fish drying took place 20 (28.2%) compared with women who slept elsewhere 43 (15.9%) *P* < 0.05. There was no difference in the educational status of the women and none of them smoked tobacco.

### 3.2. Prevalence of Anxiety and Depression among the Study Population

Table 2 shows the prevalence of psychological distress among the women. Fifty-seven women (16.6%) recorded scores >10 on the anxiety sub-scale while 51 (14.9%) recorded scores >10 on the depression subscale. Twelve women (3.5%) had combined features of anxiety and depression. In all, 96 subjects had documented features suggestive of psychological distress. Anxiety and depression were more prevalent among women with chronic bronchitis; *P* < 0.05.

### 3.3. Association between Exposure, Psychological Distress, and Chronic Bronchitis

Table 3 presents the prevalence of psychological distress among the women after separating them according to biomass smoke exposure status (<200 hour-years and >200 hour-years). In the category of women with exposure <200 hour-years, there was a higher prevalence of depression among subjects with chronic bronchitis compared with women without chronic bronchitis but not in anxiety. In the category of women with >200 hour-years of exposure, anxiety and depression were more prevalent among subjects with chronic bronchitis. This may suggest that the relationship between exposure status, psychological distress, and chronic bronchitis is not linear.

### 3.4. Predictors of Psychological Distress

Direct logistic regression was performed to examine the impact of some variables on the development of anxiety or depression. The models contained five independent variables: age, education, exposure, sleeping in the fish drying room and chronic bronchitis. The models included depression when anxiety was the dependent variable and anxiety when depression was the dependent variable. Level of exposure and the presence of chronic bronchitis made unique significant contributions to the development of anxiety after controlling for age, education, sleeping room, and depression while level of exposure, sleeping in the fish drying room, and chronic bronchitis made unique and significant contributions to developing depression after controlling for age, education, and anxiety (Table 4).

## 4. Discussion

The rationale for this study was to assess the relationship between long-term exposure to indoor air pollution from fish smoking by burning firewood, the development of chronic bronchitis, and symptoms of psychological disorder.

This study found the prevalence of chronic bronchitis among women to be 18.4%. This prevalence is higher than the 10.6% reported by Desalu et al. [14] in a survey of respiratory symptoms among women using biomass fuel for cooking in rural south-west Nigeria. Other studies outside Africa have reported the prevalence of chronic bronchitis among rural women exposed to biomass smoke ranging between 7% in Peshawar, Pakistan [24], and 12% in rural Mexico [23]. The lower prevalence of chronic bronchitis in these studies may be due to the fact that they sampled women with exposure to indoor air pollution only during domestic cooking while the subjects in this survey consisted of women with occupational exposure and possibly domestic exposure from cooking.

The HADS in spite of its name has been found to be useful in community screening for psychological distress [25–27]. In this study, we found the prevalence of anxiety to be 16.7%, depression to be 14.9%, and combined anxiety and depression to be 3.5%; in all, 28% of the women had psychological distress. This finding is in agreement with reports of 15.1% for anxiety and 13.4% for depression from a previous study of primary care patients by Michopoulos et al. using the HADS [28]. A survey by Jenkins et al. [29] in rural Nairobi, Kenya, reported the prevalence of common mental disorders to be 10.8% and this was significantly associated with older age and physical illness.

In this study, psychological distress was significantly more prevalent in women with chronic bronchitis: 30.2% of women with chronic bronchitis had anxiety compared with 13.0% of those without, while 34.9% of women with chronic bronchitis had depression compared with 10.4% without chronic bronchitis. Previous studies have documented a high prevalence of anxiety and depression in COPD patients using the HADS. Dowson et al. [21] in a study of 79 COPD patients in New Zealand found the prevalence of anxiety and depression to be 50% and 28%, respectively. Janssen et al. [30] in a cross-sectional study involving COPD patients entering a pulmonary rehabilitation programme reported a prevalence of 32% and 27% of anxiety and depression, respectively.

Most of the studies on the comorbidities of COPD have been carried out on patients with tobacco smoke exposure as the principal risk factor [4]. The comorbidities of COPD associated with chronic biomass exposure have not been extensively studied [7]. Tobacco is a biomass and as such people exposed to biomass smoke are expected to develop diseases similar to cigarette smokers. Ramírez-Venegas et al. in a study to compare the effects of biomass smoke and cigarette smoke observed that women with domestic exposure to biomass fuel combustion were at risk of developing COPD with clinical characteristics, impaired quality of life and increased mortality similar in extent to those of the tobacco smokers [31].

Of particular importance is the observation that the association between chronic bronchitis and psychiatric distress was different for different levels of exposure to indoor air pollution with the chances of developing anxiety or depression increasing with increased exposure. A similar observation was made by Wagena et al. in a previous study to evaluate the modifying effect of cigarette smoking on the association between chronic bronchitis and psychological distress: the authors concluded that the association between chronic bronchitis and psychiatric morbidity was different for smokers, past smokers, and never smokers [32].

Several mechanisms may be responsible for the observed association between chronic bronchitis, psychiatric distress and exposure to biomass smoke. Chronic bronchitis itself may be considered to be reason enough for patients to feel depressed or anxious. The presence of these psychiatric complaints may therefore be regarded as a complication of the physical complaints [33]. Increased exposure to biomass smoke has been shown to be associated with increased respiratory symptoms and chronic bronchitis [7, 17]. Several studies have shown that the risk of depression and anxiety increases by the increasing severity of respiratory complaints [34, 35].

This study has several limitations: first of all, the instrument used in assessing psychiatric morbidity; HADS is a self-administered tool but we had to employ interviewers because of the low level of education of our subjects. It is expected that subjects may underreport symptoms when interviewed but since all the subjects were interviewed by trained assistants, whatever bias that may be introduced will have a negligible overall effect. Secondly, we did not document exposure to biomass smoke from cooking. We expected that as women in a rural African community, biomass smoke exposure from domestic cooking will not be selective and will therefore not introduce bias. Thirdly, we were unable to measure directly the level of air pollution resulting from burning firewood. This would have given us the pollution levels at a particular point in time but because the outcome of interest results from long-term exposure, we were of the opinion that the cumulative exposure represented by the product of the estimated average number of hours spent smoking the fish and the duration in years of smoking fish (hour-years) was more appropriate for this. Finally, the design of the study, cross-sectional observation makes it impossible to show whether psychiatric distress precipitates chronic bronchitis or is a result of it.

In conclusion, this study has demonstrated that there is a significant association between psychiatric distress and chronic bronchitis with the biomass smoke exposure level having a modulating effect: increased exposure producing an increased risk for anxiety or depression. Psychological distress reduces a person's quality of life and also increases the force required to cope with physical illnesses. Anxiety and depression remain easily undiagnosed because of under-presentation as well as the fact that the symptoms are not very specific; it is important to consider the presence of these disorders in women with chronic bronchitis.

Recently, there has been a lot of interest in reducing indoor air pollution by providing more efficient stoves with lower potential for air pollution. The use of improved biomass stoves has been associated with a reduction in several adverse physical health outcomes [36, 37]. It is expected that a reduction in indoor air pollution related physical morbidity should lead to a reduction in psychological distress. Further studies will be required to explore such an outcome.

## Figures and Tables

**Table 1:** Characteristics of women exposed to indoor air pollution from burning firewood.

Characteristic	Women with chronic bronchitis (*N* = 63)	Women without chronic bronchitis ( *N* = 279)	Analysis*
Age in years; mean (SD)	42.9 (8.8)	39.63 (8.9)	*t* = −2.7, df = 340, *P* = 0.008
Exposure in hour-years; mean (SEM)	210.2 (14.4)	155.4 (6.3)	*t* = −3.69, df = 340, *P* < 0.001
Education			
None, no. (%)	8 (34.8)	15 (65.2)	*χ* ^2^ = 4.6**, df = 2, *P* = 0.101
Primary	47 (18.1)	212 (81.9)	
Secondary	8 (13.3)	52 (86.7)	
Exposure in hour-years			
	34 (14.5)	201 (85.5)	*χ* ^2^ = 7.8, df = 1, *P* = 0.005
	29 (27.1)	78 (72.9)	
Sleeping room^#^			
No	43 (15.9)	227 (84.1)	*χ* ^2^ = 5.6, df = 1, *P* = 0.18
Yes	20 (28.2)	51 (71.8)	

*Comparison of women with chronic bronchitis with women without chronic bronchitis; **Likelihood ratio.

^#^Sleeping in the same room where the fish smoking was carried out.

SD: standard deviation.

**Table 2:** Prevalence of anxiety and depression and the unadjusted odds ratio for the association between chronic bronchitis and psychological distress.

	Anxiety *N* = 57 (%)	*χ* ^2^	*P*	OR (95% CI)	Depression *N* = 51 (%)	*χ* ^2^	*P*	OR (95% CI)
Women with CB*	19 (30.2)	10.1	0.001	2.7 (1.4–5.2)	22 (34.9)	24.4	<0.001	4.6 (2.4–8.8)
Women without CB	38 (13.6)				29 (10.4)			

CB: chronic bronchitis.

**Table 3:** Prevalence of anxiety and depression and the unadjusted odds ratio for the association between chronic bronchitis and psychological distress according to exposure level.

Exposure (hour-years)	Anxiety *N* = 57 (%)	OR (95% CI)	*P*	Depression *N* = 51 (%)	OR (95% CI)	*P*
<200 hour-years						
Women with CB*	5 (14.7)	1.11 (0.4–3.1)	0.8	10 (29.4)	3.8 (1.6–9.0)	0.004
Women without CB	27 (13.4)			20 (10.0)		
>200 hour-years						
Women with CB	14 (48.3)	5.7 (2.2–15.0)	0.001	12 (41.4)	5.4 (2.0–15.0)	0.001
Women without CB	11 (14.1)			9 (11.5)		

CB: chronic bronchitis.

**Table 4:** The adjusted odds ratio for the association between individual study variables and psychiatric morbidity among women exposed to biomass smoke.

Parameter	Anxiety		Depression	
	OR (95% CI)	*P*	OR (95% CI)	*P*
Age	0.9 (0.74–1.01)	0.27	0.99 (0.95–1.03)	0.68
Education	1.2 (0.61–2.2)	0.65	0.7 (0.17–2.7)	0.61
Exposure	1.02 (1.01–1.1)	0.003	1.03 (1.10–1.08)	0.04
Sleeping* room	1.8 (0.88–3.5)	0.11	2.7 (1.32–5.3)	0.006
CB	2.3 (1.2–4.7)	0.02	3.6 (1.8–7.3)	<0.001

CB: chronic bronchitis.

*Sleeping in the same room where fish smoking was carried out.
